# Conceptualizing controversies in the EU circular bioeconomy transition

**DOI:** 10.1007/s13280-022-01730-2

**Published:** 2022-03-23

**Authors:** Jan R. Starke, Tamara A. P. Metze, Jeroen J. L. Candel, Catrien J. A. M. Termeer

**Affiliations:** Department of Social Sciences, Public Administration and Policy Group, Wageningen University & Research, 6700 EW Wageningen, The Netherlands

**Keywords:** Biorefinery, Circular bioeconomy, Discourse coalition, European Union, Policy controversy, Sustainability transition

## Abstract

The transition towards a circular bioeconomy (CBE) in the European Union is not without contestation. In particular, research has highlighted potential trade-offs of the large-scale production of bio-resources, for instance with environmental quality goals. To date, however, it remains underexplored in the CBE literature how controversies develop throughout a transition process. To address this gap, this paper explores where controversies are situated in a transition, how they change throughout, and how they influence the transition process. First, we suggest that controversies can be situated on and between different system layers within a transition. Second, we offer an explanation of how controversies evolve, as actors confirm, integrate, disintegrate and polarize underlying storylines. Third, these controversies can have both productive and unproductive outcomes while they unfold throughout a transition. We illustrate this understanding with the example of biorefineries as CBE key technology and discuss a research agenda on controversies in sustainability transitions.

## Introduction

Shifting towards a circular bioeconomy (CBE) is cherished widely within the European Union (EU) and beyond as an answer to current challenges, such as the depletion of fossil resources, climate change and the environmental impact of human production and consumption (Meyer 2017; Priefer et al. 2017; European Commission 2018; D’Amato et al. 2020). The road towards a CBE constitutes an ongoing shift from the current—predominantly linear—extract-use-dispose logic of production based on fossil resources, towards an envisioned circular system, based on sustainably sourced renewable resources such as plants, fungi and algae (McCormick and Kautto 2013; Bugge et al. 2016; Kirchherr et al. 2017). We understand this change process as a sustainability transition, i.e. a large-scale societal shift from a normatively undesired (unsustainable) state towards a desired (sustainable) one (Markard et al. 2012; Loorbach et al. 2017; Köhler et al. 2019).

Despite high expectations for the CBE, previous research has recognized that the CBE transition is not without contestation. The large-scale production of bio-resources as industrial feedstock can entail trade-offs, for example regarding biodiversity conservation, environmental quality, and resulting human welfare (Gawel et al. 2019; Buchmann-Duck and Beazley 2020). Pursuing a CBE based on economic growth and increased production of bio-resources is criticized for not addressing problems of unsustainability, for example the question of whether some humans consume more than our planet can sustain (Vivien et al. 2019). Furthermore, conflicts have arisen about land available for uses that compete with bio-resource production, for instance food production, biodiversity conservation or recreation (Muscat et al. 2020). As bio-resources are scarce, their distribution as feedstock for different purposes (for example, the production of materials versus the production of electricity or warmth) is conflict laden (Meyer 2017). In addition to conflicts about the distribution of available bio-resources, conflicts also arise about where to locate production sites such as large biorefineries (Serrano-Hernandez and Faulin 2019). Biorefineries are a key technology in the CBE transition because they convert bio-based resources into materials such as chemicals, plastics or feed products (Cherubini 2010). Controversies around biorefineries serve to illustrate our conceptual arguments. Examples of controversies in the CBE transition with relevance for biorefineries include food-feed-fuel (Muscat et al. 2020), green growth versus degrowth (D’Alessandro et al. 2020), globalization versus regionalization (Priefer et al. 2017, pp. 12, 13) or techno-optimism versus techno-scepticism (Arancibia 2013; McCormick and Kautto 2013).

Policy controversies (in short: controversies) are a particularly intractable form of conflict. “A *conflict* exists whenever *incompatible* activities occur” (Deutsch 1973, p. 10, original highlighting). Importantly, activities do not actually need to be incompatible; ideas about their incompatibility are sufficient to incite conflict (Deutsch 1973). Controversies are situations in which involved actors “see issues, policies, and policy situations in different and conflicting ways that embody different systems of belief and related prescriptions for action” (Schön and Rein 1994, p. xviii). These "underlying structures of belief, perception, and appreciation" (Schön and Rein 1994, p. 23) are called frames. Consequently, we use the term *conflict* when referring to incompatibilities between actors in a broad sense, and the term *controversy* when referring to intractable framing conflicts. Controversies are particularly relevant in the EU CBE transition, as both of the concepts forming the transition’s goal, *circularity* and *bioeconomy*, are contested (Bugge et al. 2016; Kirchherr et al. 2017; Bauer 2018) and thus prone to conflicting interpretations.

As we will show in the “Current perspectives on conflict in the CBE literature” section, it remains underexplored how controversies change throughout the CBE transition and how they influence the transition process. We differentiate two overarching perspectives on conflict in the state-of-the-art CBE literature: (i) “conflict as design fault”, which approaches controversies as an optimization problem that needs to be resolved, and (ii) a “framing conflicts” perspective, which acknowledges that controversies are an inherent element of the CBE transition and identifies actor groups around conflicting frames. We, however, show that the former struggles to explain why controversies reappear during a transition process despite resolution approaches, whereas in the latter, it remains unclear how these frames change dynamically throughout a transition. In this contribution, we therefore aim to advance the understanding of how controversies develop throughout the transition towards a CBE in the EU. Moreover, we propose conceptual entry points with the ambition to further explore how these developments of controversies influence the transition process.

We argue that current perspectives on controversies in the CBE transition could be advanced in three regards. First, we situate controversies within the transition process. We argue that actors move controversies through different loci on and between multiple system layers: micro, meso, and macro (“Situating controversies in the transition process” section). Second, we problematize how controversies change throughout a transition. Groups of actors involved in these controversies continuously change the storylines they tell to communicate their understanding of what is problematic and how these problems should be solved (“Changes of controversies due to dynamic storylines” section). Third, we highlight the outcomes of controversy changes on the transition process. Controversies can indeed develop in unproductive ways and paralyze the transition. However, productive changes of controversies can add to a more reflexive, innovative and democratic form of CBE transition (“Unproductive and productive outcomes of controversies” section). Subsequently, we discuss the implications of our conceptual work for both researchers and practitioners and sketch a research agenda on controversies in sustainability transitions (“Discussion and research agenda” section).

## Current perspectives on conflict in the CBE literature

Despite acknowledging the relevance of conflict, the ways in which controversies develop as well as their outcomes on an unfolding transition process remain largely underexplored in the state-of-the-art literature on the CBE. For the discussion of the literature in this section, we searched Scopus and Google Scholar for publications (peer-reviewed and grey literature) that use the terms “*bio-based economy”, “*biobased economy”, “*bioeconomy”, “circular economy” or “biorefin*” in their title, keywords, or abstract. We selected publications based on the number of citations, while also including recent publications, and for containing the terms “conflict*”, “controvers*” or “accepta*”. All references were checked for additional relevant publications. Subsequently, we synthesized the literature into two overarching perspectives on the role of conflicts and controversies in the CBE transition: a perspective on conflict as a design fault of novel technologies and supply chains and a perspective focusing on framing conflicts to (de)legitimize transition pathways and visions.

The two perspectives differ in their ontological positions. Authors adopting the perspective of conflict as design fault regard conflict as an objective problem to be solved to advance the CBE transition. Knowledge is understood as a tool to solve conflicts. In contrast, the framing conflicts perspective underlines that conflicts are socially constructed and an inherent element of transitions, which cannot be solved for good. Scholars adhering to this perspective highlight that actors frame knowledge divergently or may draw on different sources of knowledge. Knowledge can therefore also be a source of conflict (Metze 2018). In this article, we contribute to both perspectives by contextualizing controversies as an intractable type of conflict in the transition process and conceptualize how such controversies change throughout a transition.

### Conflict as design fault

Particularly in techno-economic contributions, conflict is understood as a negative societal effect resulting from an incongruence of interests between actors, which can and should be resolved. In the CBE literature, techno-economic analyses are frequently carried out to identify the societal effects of key technologies in the CBE transition (e.g. Kokkinos et al. 2018; Vyhmeister et al. 2018; Serrano-Hernandez and Faulin 2019; Zetterholm et al. 2020).

Novel biorefinery designs compete on scarce bio-resources for the production of materials with other purposes, such as bio-resources for animal feed or energy production (Muscat et al. 2020). These distributional conflicts are assumed to be overcome by tools such as supply chain optimization (Zandi Atashbar et al. 2018) and appropriate production site planning (Santibañez-Aguilar et al. 2014). Considering biorefinery supply chains as an optimization problem assumes that negative social and economic impacts can be prevented by selecting cost-efficient designs and plant locations. For example, Serrano-Hernandez and Faulin (2019) establish a calculation method for the optimal location of a large-scale biorefinery in Northern Spain. They propose a location strategy based on feedstock purchase, transport, and storage to pinpoint a cost-optimal location for a new biorefinery (Serrano-Hernandez and Faulin 2019, pp. 89, 90). The authors claim that, based on this generated knowledge, “decision makers could take advance in next negotiation processes with farmers” (Serrano-Hernandez and Faulin 2019, p. 91). It is thus assumed that a rational positioning decision mitigates conflicts with local farmers and helps create societal acceptance of new installations.

In this perspective, conflicts stem from unintended, negative sustainability impacts of novel technologies that diminish societal acceptance. For example, Souza et al. (2018) recognize that different biorefinery set-ups for producing biofuels from sugarcane in Brazil lead to different impacts on society, for instance varying levels of job creation and different numbers of accidents. Also Yao and Tang (2013, p. 1707) conclude that “improved acceptance and conscientious understanding among the public” need to accompany the development of new renewable chemicals and polymers. Furthermore, Moretto et al. (2020, p. 5) regard societal acceptance in addition to legislative barriers as obstacles for products from an urban waste biorefinery in Italy and suspect consumer values such as “green self-identity” and “awareness of recycling” as factors affecting the acceptance of bio-based products. Consequently, analysts sometimes regard conflicts as bad news, impeding societal acceptance and transition support (e.g. Peck et al. 2009; Arancibia 2013; Gawel et al. 2019). However, controversies can also be beneficial by stimulating decision makers to learn from different perspectives and thus achieve a more reflexive form of CBE transition (Cuppen 2018; Metze 2018).

Conflicts arising from the lack of technology acceptance are then implicitly assumed to be prevented by design choices based on advanced lifecycle assessments. For example, Sillero et al. (2021) compare six process design routes to valorize almond shells in terms of their overall environmental impacts. The explicit goal is to identify “the most suitable one for large-scale valorisation” (Sillero et al. 2021, p. 749). According to this view, engineers can thus ‘out-design’ conflicts by a smart appreciation and subsequent limitation or elimination of negative impacts.

Although such assessments are certainly useful for estimating and comparing impacts of different process designs ex ante, understanding conflicts solely as a design fault or optimization problem struggles to grasp the complexities of policy controversies, though. This is because conflict is regarded as a *static* barrier that needs to be overcome to engineer technology acceptance. However, controversies are *dynamic*, popping up again and again during a transition (Yuana et al. 2020). What is more, controversies are particularly intractable to resolution approaches such as providing information about rational benefits or negotiation (Schön and Rein 1994; Hisschemöller and Hoppe 1995; Van Eeten 1999). Conflicting actors use this form of fact knowledge to increase the credibility of their previously established arguments (Metze 2017; Wolf and Van Dooren 2017), thus use fact knowledge politically. Hence, controversies cannot be overcome by generating objective fact knowledge, for instance in the form of impact assessments.

Moreover, the cost-optimal location planning of large new installations is not always the most accepted and thus least controversial choice, given that the local population could introduce new concerns and problem understandings that have not been considered before. The local population might perceive the costs to them (e.g. facility-related traffic, emissions, land use) as disproportionately high compared to the benefits for the broader region (e.g. employment, progress in the CBE transition) and thus engage in a not-in-my-backyard argumentation (see Hisschemöller and Hoppe 1995). Scholars and engineers should thus be careful in assuming that an objective calculation of cost-efficient positioning strategies for biorefinery facilities or ‘out-designing’ aspects that experts regard as controversial translates directly into local acceptance. Positioning a biorefinery is not purely a cost-rational act based on objective calculations, but also political. Controversies thus need to be understood in the context of broader transition processes. However, it seems as yet unclear how controversies are contextualized in a transition, thus where and why controversies continue to arise again and again in transition processes.

### Framing conflicts

Contributions applying a framing conflicts perspective not only regard conflicts as interest incongruencies, but also clarify that conflicts are rooted in different framings of both the problem and proposed solutions. Empirically, in transitions, problem definitions and connected solutions are often formulated in policy transition visions and pathways. The transition *vision* is the goal of the transition process, for example a CBE as outlined in the EU Bioeconomy Strategy. In addition to conflicts about what the CBE vision should entail, conflicts arise on the right way to get there, thus on competing ideas about *pathways* to achieve the vision (cf. Geels and Schot 2007). Examples of pathway elements in the CBE transition include the form of technology to use, how to consider sustainability trade-offs, or in what way stakeholders should participate in vision definition and pathway selection (Priefer et al. 2017).

In this line of reasoning, visions and pathways on how to achieve a bioeconomy are framing conflicts in which particular problem perceptions and solutions are legitimized or delegitimized by different groups of actors. These loosely connected networks of actors promote conflicting storylines to organize political support. Storylines are socially constructed communicative devices to “condense large amounts of factual information intermixed with the normative assumptions and value orientations that assign meaning to them” (Fischer 2003, p. 87).^1^ Social scientists in the CBE literature have focussed on identifying these conflicting storylines and the actor groups advocating them (e.g. Bauer 2018; Peltomaa 2018; Giurca 2020; Sanz-Hernández et al. 2020; Simoens and Leipold 2021).

For example, Peltomaa (2018) identifies five storylines in an analysis of Finnish newspaper articles on the bioeconomy during the periods 2010–2011 and 2015–2016: a biotechnology-centred bioeconomy, a resource-centred bioeconomy, an agroecological bioeconomy, bioeconomy as skilfulness, and a climate-change-centred bioeconomy. The storylines are reproduced by different actor groups. For instance, whereas dominant storylines seem to be advocated by industrial actors, experts and politicians, the agroecology storyline is voiced by “farmers, citizens, or activists” (Peltomaa 2018, p. 10). Although Peltomaa (2018, p. 12) acknowledges in his discussion that storylines “are not stable but change over time”, the study’s focus was to identify stable storylines and advocating groups of actors. The precise mechanisms and actor motivations leading to changes of these groups had to remain a black box.

This perspective acknowledges the intractability of controversies in the CBE transition by stressing that controversial aspects are defined differently by different actors. However, *changes* of controversies throughout a transition process are underexplored (see Leipold 2021 for a notable exception on EU circularity policies). Although we acknowledge the relative stability of actor groups, we add to this perspective by explicitly conceptualizing how and why storylines and associated actor groups change over time, particularly in long-term controversies, and what this means for the overall transition process. In controversies, new actors enter the group, others leave, the underlying storyline is continuously re-defined, and new groups develop. We therefore propose to highlight the dynamics of these discursive conflicts by regarding underlying storylines as continuously evolving. In this sense, we first situate controversies in the transition process, then explain how controversies develop due to changes in underlying storylines, and finally reflect on the outcomes of changing controversies on the overall transition process.

## Situating controversies in the transition process

**Fig. 1:**
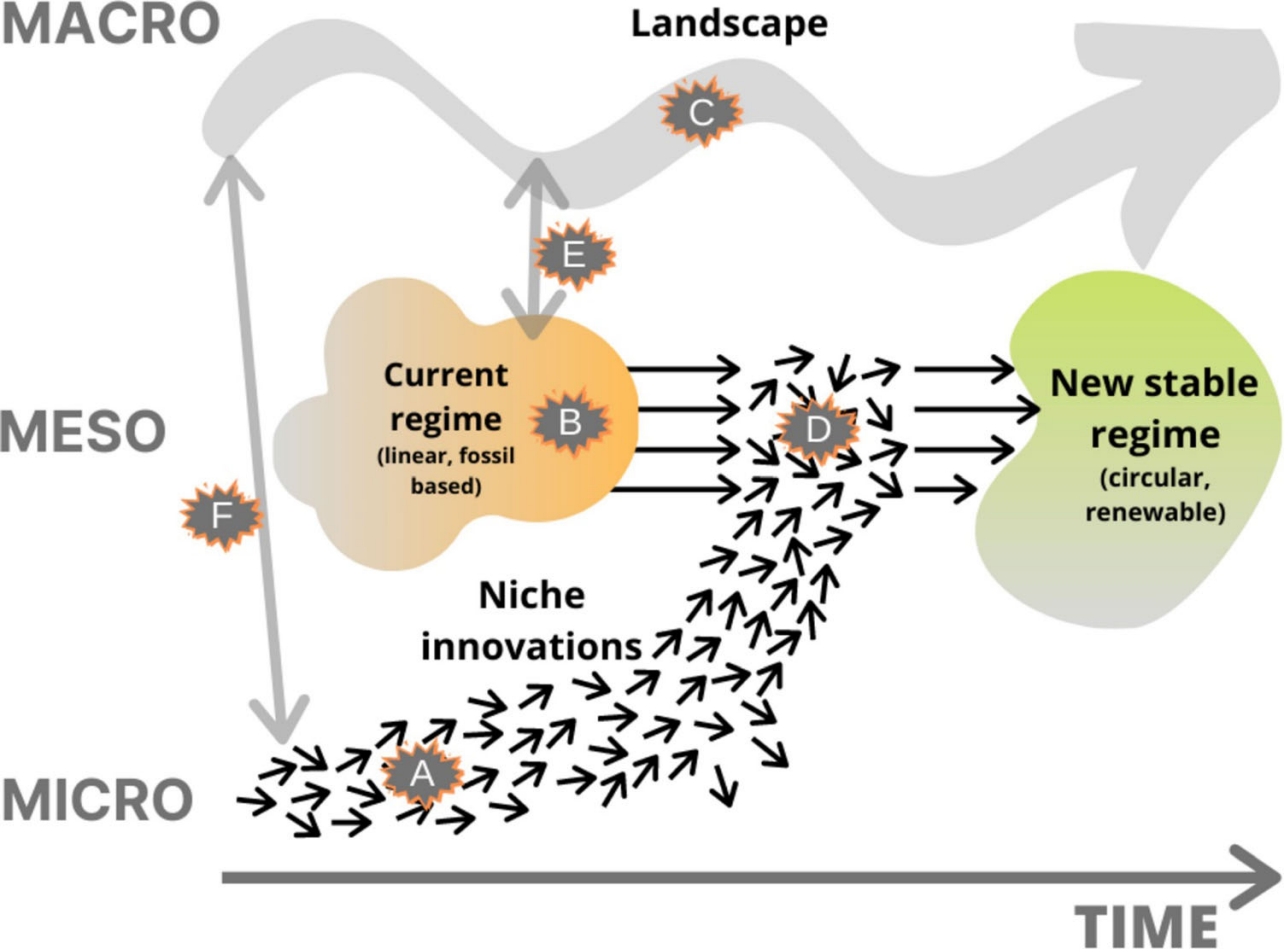
Loci of transition controversies on and between different system layers, after Geels (2002), Geels (2005), Loorbach et al. (2017), Van Der Minne et al. (2021)

To assess how controversies develop throughout a transition, we first situate controversies within the transition process. We argue that controversies arise in different forms on and between different system layers during a transition. The much-used multi-level perspective (MLP) on transition processes (Geels 2002, 2005, 2019) distinguishes three system layers: micro, meso, and macro. According to this understanding, transitions advance thanks to interactions of micro-level niches, meso-level regimes, and the macro-level landscape. In our case, *macro-level* pressure (the—perceived—need for fossil-free alternatives for depleting fossil resources) in combination with alternative options from *micro-level* niches (novel biorefineries) lead to a change from a fossil-based *meso-level* regime (linear, fossil-based production) towards a new regime (CBE). In line with this understanding, we distinguish loci for CBE transition controversies on and between micro, meso and macro levels, Fig. 1. Controversies are contextualized in these loci, which are specific locations in a transition process with particular involved (groups of) actors, frames and communication rules.

The micro-level locus (A in Fig. 1) hosts controversies in small-scale, detailed and exclusive settings, for example expert discussions on novel biorefinery set-ups. Involved actors are “outsiders and entrepreneurs […] ‘below the surface’ of incumbent regime actors” (Geels 2011, p. 498). Examples include a group of independent scientists working on a novel biorefinery design or an off-grid, self-sustaining community thinking about new ways of utilizing organic waste. Micro-level controversies concern alternative, regime-challenging ways of thinking, doing and organizing (Van Der Minne et al. 2021). Involved frames can be divergent from one another but commonly deviate from state-of-the-art frames. An example of a micro-level controversy is a discussion between two expert groups working on alternative biorefinery designs: decentralized, small-scale biorefineries versus centralized, large-scale integrated biorefineries.

The meso-level locus (B in Fig. 1) hosts controversies in the bureaucratic setting of current rules and infrastructure. One example is the permit process to locate a new biorefinery. Meso-level controversies concern the dominant way of thinking, doing and organizing (Van Der Minne et al. 2021) and gradual adaptations of the status quo. Involved actors can be (departments of) companies in fossil sectors and their industry organizations (Geels 2004), (units of) ministries (Verbong and Geels 2007), municipal civil servants or administrators of established infrastructures such as the gas grid. The set of involved actors is thus rather limited, actors are well-established, have high stakes, and are connected to the dominant set of frames. Regime controversies concern the distribution of resources and how to gradually adapt to pressures from both landscape and niches. Hence, these controversies can entail, for instance, the radicality as well as the technical or economic feasibility of such adaptations.

The macro-level locus (C in Fig. 1) concerns landscape developments, which are “cultural changes, demographic trends, [and] broad political changes” (Geels 2002, p. 1262), among other long-term trends. In the CBE transition, macro-level developments include, for instance, diminishing fossil resources and the resulting demand for fossil-free alternatives. Macro-level controversies concern interpreting the need for action stemming from these landscape developments. One example is the shaping of the EU Bioeconomy Strategy. When controversies surface on the macro-level, not only are direct stakeholders involved, but also the broader public becomes engaged in these discussions. Involved sets of actors and frames are therefore wide and divergent.

The different configurations of actors and associated frames present *across* these loci are of particular interest for controversies. For example, whereas engineers might develop a new biorefinery design on the micro level, policymakers craft strategic decisions on the future of the CBE in the EU on the macro level, and the installation ultimately has to be located in a municipality, concerning the meso level. As the loci are interlinked, controversies can also be located between the different system layers. Most prominently, micro-meso controversies (D in Fig. 1) are controversies between niche innovators and regime incumbents (e.g. Hess 2014; Leipprand and Flachsland 2018). Regime actors typically highlight current hindering regulations or high costs, whereas innovators argue that their innovations are a better way to handle landscape pressure. For example, a controversy could develop between an innovative micro-level expert team proposing a biorefinery using genetically engineered algae as feedstock and facing meso-level regime regulations impeding the use of this feedstock. Such controversies could result in a delegitimization and consequently a destabilization of the current regime (Bosman et al. 2014). Macro-meso controversies (E in Fig. 1) concern incongruencies between the need to adapt the regime because of landscape pressure and regime lock-ins impeding this adaptation. Regime actors, for example, might favour small adaptations (e.g. blending biofuels from the novel algae-based biorefinery into conventional fuels), whereas the landscape pressure might require more radical actions (e.g. banning internal combustion engines and thus requiring a different product from the biorefinery). Macro–micro controversies (F in Fig. 1) arise from incongruencies between micro-level innovations and macro-level pressures. For instance, a macro-level EU strategy could point out the risks of using genetically engineered algae and strive to use sustainably sourced wood as a feedstock, whereas micro-level engineers might assess the risks as insignificant compared to the economic and technical benefits of using genetically engineered algae.

Controversies might be more visible in some loci than in others at different junctures. For example, the algae controversy could be salient between micro- and macro-level actors in the design phase of a biorefinery and pop up later in the form of meso- and macro-level citizen concerns about locating the new facility. As a result, controversies are intractable because actors move them through the different system layers and controversies thus reappear in different loci.

## Changes of controversies due to dynamic storylines

While controversies move through the different loci within a transition process, actors adjust underlying storylines, resulting in controversy changes. Actors involved in controversies do not act in isolation, but rather form groups around similar storylines. For example, Leipold (2021) argues that current circular economy storylines in the EU are shaped by a joint coalition of business- and environment-oriented parts of the European Commission. Controversies evolve within a transition process due to interactions between actor groups and storylines. This means that involved actor groups adjust underlying storylines throughout the transition process. As a result, actor groups may grow, shrink, merge or fall apart.

We conceptualize these actor groups as *dynamic discourse coalitions* (Metze and Dodge 2016) around common storylines, which involved actors reproduce and shape. Discourse coalitions are “defined as the ensemble of (1) a set of story-lines; (2) the actors who utter these story-lines; and (3) the practices in which this discursive activity is based” (Hajer 1995, p. 65). Discourse coalitions gather around shared storylines in congruence with underlying discourses (Hajer 1995). A discourse is “a specific ensemble of ideas, concepts, and categorizations that is produced, reproduced, and transformed in a particular set of practices and through which meaning is given to physical and social realities” (Hajer 1995, p. 60). Discourse coalitions sponsor a shared interpretation of a social reality, which they continuously reinvent and thus also shape (Fischer 2003).

Coalition building can be both strategic and unintentional. Actors are not always conscious of the frames that they apply and can unintentionally form discourse coalitions with actors applying similar frames. However, by reflection, actors can become aware of the frames channelling their thinking and learn to adjust them, i.e. to reframe (Schön and Rein 1994). Actors in different discourse coalitions frame knowledge and experiences divergently because they make sense of new information and select, name, and categorize aspects strategically to build their storylines (Van Hulst and Yanow 2016). Actors therefore have agency in framing, meaning that they can highlight selective aspects of reality strategically. Hence, actors use storylines to legitimize a particular vision as well as pathways with connected tools, strategies and interventions to achieve this vision (cf. Hajer 1995; Fischer 2003; Bauer 2018). For example, a challenging coalition can successfully delegitimize dominant regime storylines, contributing towards regime destabilization (Bosman et al. 2014).

Storylines and surrounding discourse coalitions develop throughout the transition process. More specifically, discourse coalitions change over time through processes of *confirmation* (strengthening of a storyline), *integration* (connection of storylines), *disintegration* (contestation of a storyline within the discourse coalition itself) and *polarization* (reconfirming the differences in competing discourse coalitions’ storylines) (Metze and Dodge 2016, p. 4). Hence, storylines are not designed once and then remain stable throughout the transition period; rather, actors continuously reproduce storylines and produce new ones. Because of changes in storylines, the surrounding discourse coalitions are also in constant flux: new actors join the coalition along the transition process, others leave it.

**Fig. 2:**
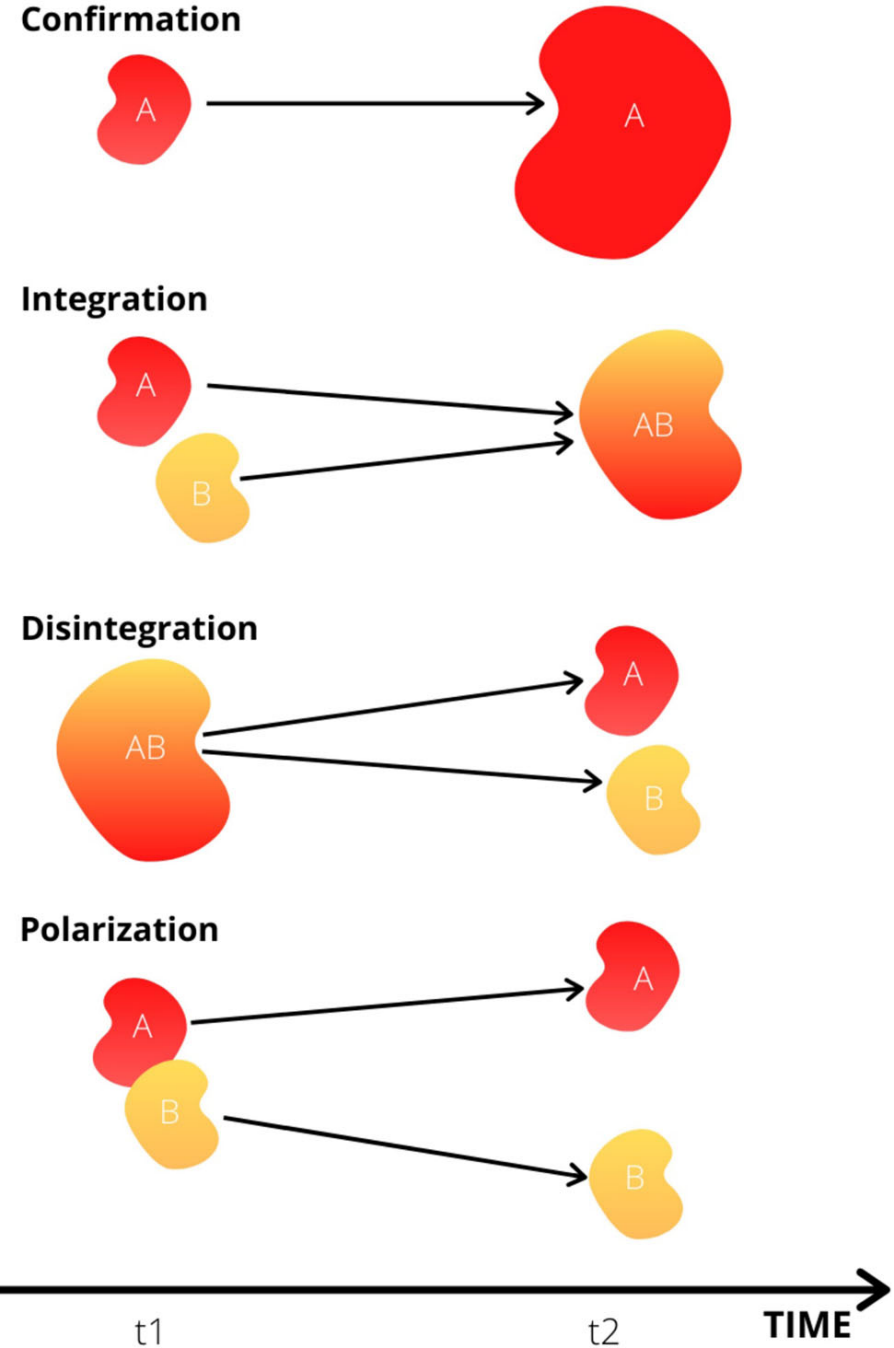
Four possible shifts in dynamic discourse coalitions over time

Figure 2 illustrates processes whereby dynamic discourse coalitions shift over time. First, a discourse coalition can *confirm* its underlying storylines. For instance, a coalition around policymakers, scientists, and companies, which favours large-scale, central biorefineries, produces new scientific reports that underlines their storyline that this form of biorefinery is indeed the most cost-efficient, economically feasible, and thus desirable form. As a result, the coalition can grow, for example in number, resources, or the persuasiveness of their storylines.

Second, two separate discourse coalitions can *integrate* their storylines and merge. For example, coalition A reproduces the storyline that non-food (e.g. lignocellulosic) feedstock biorefineries are more accepted than biorefineries using food crops. Coalition B promotes the storyline that supply structures with a central, large-scale biorefinery are more feasible than supply structures with decentralized, small-scale biorefineries. Integrating these storylines, coalition AB sponsors the storyline that a central biorefinery using lignocellulosic feedstocks is the most feasible and accepted design.

Third, discourse coalitions can *disintegrate* over time. For example, coalition AB later splits into two coalitions, coalition A promoting marine feedstocks and coalition B promoting forest-based ones.

Fourth, actors can work to *polarize* discourse coalitions. In our example, the two initially close and even overlapping discourse coalitions A and B depart from one another. Coalition A (promoting algae) starts to make moral claims in public discussions about coalition B (promoting wood), arguing that using wood contributes to the destruction of forests and is therefore morally inferior. Polarization in controversies can even result in misinformation and undermining scientific evidence because involved actors instrumentalize new knowledge to legitimize a preferred transition pathway or to support the status quo. Competing coalitions interpret new knowledge so that it corresponds with their established frames. Each discourse coalition therefore creates its own interpretation of new evidence. Instead of providing an objective solution, new evidence can thus also lead to new controversies on how to interpret this new fact knowledge, hence generating new polarization (Metze 2018).

## Unproductive and productive outcomes of controversies

Controversies evolve during the transition process in both unproductive and productive ways. These developments affect the inter-personal relationships of actors shaping both transition vision and pathways in dynamic discourse coalitions, while the transition process develops. Therefore, we propose criteria on how to differentiate unproductive and productive evolutions of controversies while they unfold in a transition process.

On the one hand, controversies can evolve in an unproductive way. Actors can escalate a conflict from a substantial level (disagreement on content, for example on the question of what is the biorefinery set-up with the least CO_2_ emissions) to a procedural level (who defines what is counted as CO_2_ emissions?) and further to the level of inter-personal relations (accusations of polishing CO_2_ assessments) (cf. Wolf and Van Dooren 2021). This form of escalation leads to an increase of distrust between conflicting actors (Wolf and Van Dooren 2021). Increasing distrust can then lead to a deterioration of relations (cf. Deutsch 2014), resulting in conflicting actors viewing “themselves as moral and their opponents as immoral and unreasonable” (Kriesberg and Dayton 2017, p. 158). This can permanently damage inter-personal relationships and is thus likely to jeopardize future intents to rebuild trust and reinstall collaboration (Wu and Laws 2003). Another sign of a controversy becoming unproductive is when key actors manage to actively exclude actors with a different perspective from decision-making processes. Moreover, actors talking past each other without regarding the arguments of their adversaries is an unproductive form of controversy (cf. Van Eeten 1999). Importantly, this is not a stepwise development. For instance, a deterioration of relations does not always precede the exclusion of others. Moreover, the different examples are not necessary ordered hierarchically in order of their magnitude. For example, talking past each other is not necessarily a more intense evolution than the exclusion of others.

On the other hand, controversies are not always bad news. Controversies can evolve in productive ways, meaning that they improve the CBE transition process. Controversies can stimulate learning (cf. Cuppen 2018; Metze 2018) and thus obtain innovative potential. Actors engaged in transition processes generate knowledge of many kinds, for example new alternative options to handle macro-level pressure to act and new empirical experience of the micro-level approaches that do or do not work. In addition to technological innovations, such as biorefineries, these can also be social innovations, for instance novel transformative storylines (cf. Avelino et al. 2019; Wittmayer et al. 2019). Actors can also learn by reflecting on their frames and adjust them, if necessary (Schön and Rein 1994). This form of frame innovation can contribute new perspectives to intractable controversies, shake them up, and thus help overcome stalemates. Moreover, new actors become aware of one another during the course of a transition, meet one another, begin to collaborate and become connected. Controversies can thus bring together previously unconnected actors in new groups with new resources and new power relations. Hence, productive controversies can add to new dynamics. Moreover, controversies can motivate actors to voice legitimate concerns, which have been previously overlooked. These additions of also emotional and value-based aspects increase the knowledge base for decision-making in the transition process. Furthermore, having experienced successful collaborations despite their different perspectives, actors can develop increasing trust in each other, what is in turn a fruitful ground for new collaborations (Kriesberg and Dayton 2017).

## Discussion and research agenda

Controversies in the CBE transition cannot be ‘out-designed’. We have provided conceptual steps towards understanding how these controversies change during a transition and how controversies shape a transition process. This understanding provides opportunities for both analysts and practitioners in the environmental sciences in at least three regards. Based on these opportunities, we suggest a multidisciplinary research agenda on the changes of controversies in sustainability transitions.

First, understanding transition controversies as potentially productive and exploring criteria for such beneficial evolutions of controversies in a transition process provides a basis for policymakers to guide controversies towards more productive forms. Corresponding interventions include exploring all relevant dimensions of key innovations such as biorefineries, in particular value concerns in addition to technical and economic aspects. In this way, policymakers can encourage exchanges between different perspectives to foster learning and mitigate unproductive evolutions of controversies. In practical terms, such interventions would stimulate deliberations on transition visions and pathways, the design of technical and social innovations, but also to reflect on the necessity of particular technologies to achieve the goals of the overall transition. Importantly, ill-designed interventions to manage conflicts can lead to more distrust (Wolf and Van Dooren 2021) and thus give rise to unproductive forms of controversy. Future work thus needs to be done on the careful design and testing of governance interventions to achieve more productive forms of controversy.

Second, our conceptualization provides a more context-sensitive understanding of CBE transition politics. This is in line with a call for a more contextualized appreciation of transition conflicts (Avelino 2021) as well as to better regard societal conflicts on the road towards a CBE (Vogelpohl and Töller 2021). Tracing back the development of current coalition constellations and underlying storylines creates a more complete picture of controversies in the CBE transition. This temporal contextualization provides analysts with a lens to assess how controversies have developed, what controversial aspects of the CBE transition have surfaced before and could reappear in the future. Put practically, an analysis of shifting discourse coalitions provides insight into *how* ideas (storylines binding discourse coalitions together) change in a transition process, where these ideational changes affect policymaking (shifting legitimization of transition vision and pathways), and how this translates into changes in micro-level innovations and policies. Deeper, actor-level examinations could focus on *why* actors adjust their ideas, what strategies they pursue and the capacities of actors to institutionalize their ideas. A next step would be to empirically connect the different dimensions of our conceptual advances: what dynamics of discourse coalitions shifting through the different loci explain whether a controversy evolves in unproductive or productive ways? Moreover, future empirical research could apply our conceptualization to identify different controversies and their dynamics in other sustainability transitions. We have illustrated our conceptual advances by examples from the literature on biorefineries in the EU CBE transition. Other contexts might yield further or different controversy aspects. Therefore, the conceptualization should also be applied in different contexts, for example in neighbouring energy, food, or water management transitions. Moreover, we suggest applying the conceptualization on different scales, from the international level to nations, regions, municipalities or organizations.

Third, conceptualizing controversies as inherent element of a transition contributes to a better understanding of the role of emotions and values in conflicts. These aspects should not be neglected in techno-economic assessments and the design of technical innovations in the context of transition processes. This is also highlighted in discussions on responsible research and innovation (cf. von Schomberg 2013). Understanding changes of controversies in the context of a transition helps to illuminate the discursive dimension of responsible research and innovation, as is recently called for (Jakobsen et al. 2019). A good example of the inclusion of value-based aspects in technology design is to co-design biorefinery technologies in value sensitive design processes (Palmeros Parada et al. 2020). Analyzing value-based aspects could be key in finding out why some controversies are gridlocked or smoulder under the surface only to pop up repeatedly during a transition. Seemingly logical and rational design choices (for example the chosen feedstock, which was most suitable in technical assessments) could become controversial later on in the municipality where the installation is envisioned to be located. Future research could design methods to integrate meso- and macro-level concerns already in micro-level design steps. A practical example could be to let stakeholders craft design principles for a novel biorefinery design.

In summary, conceptualizing the changes of controversies in the CBE transition is a first step towards designing governance interventions to stimulate productive forms of controversy. This is a fruitful way towards a more democratic, inclusive and responsible form of CBE transition.

## Footnotes

1 Some authors use the concept of storylines, while others refer to narratives. For the sake of conceptual coherence, we do not differentiate between storylines and narratives and uniformly use the concept of storylines.
